# An analytical framework to assess SDG targets within the context of WEFE nexus in the Mediterranean region

**DOI:** 10.1016/j.resconrec.2020.105205

**Published:** 2021-01

**Authors:** Anna Malagó, Sara Comero, Fayçal Bouraoui, Cevza Melek Kazezyılmaz-Alhan, Bernd Manfred Gawlik, Peter Easton, Chrysi Laspidou

**Affiliations:** a European Commission, Joint Research Centre (JRC), 21027 Ispra, Italy; b Department of Civil Engineering, Istanbul University- Cerrahpaşa, Avcılar Campus, 34320 Avcılar, Istanbul, Turkey; c independent environmental consultant at Easton Consult SPRL, Brussels, Belgium; d Department of Civil Engineering, University of Thessaly, 38334 Volos, Greece

**Keywords:** Water-Energy-Food-Ecosystems (WEFE) nexus, Case studies, Mediterranean region, Sustainable Development Goals (SDGs), analytical framework

## Abstract

Understanding the complex relationships amongst Water, Energy, Food and Ecosystems (WEFE nexus) together with the achievement of Sustainable Development Goals (SDGs) is critical for the development of a sustainable and secure future in the Mediterranean area. In this study, we analysed 29 case studies across the Mediterranean region which describe potential success stories for the implementation of good nexus practices. We developed an analytical framework for investigating the impacts on 15 SDG targets and we also explicitly quantified the magnitude of interconnection of nexus pillars with SDGs. Our findings showed that renewable energies have a predominant role on sustainability. Moreover, to achieve the highest positive impacts on economy, environment and society, it is necessary to ensure that both people and ecosystems benefit from a minimum amount of goods/qualities as expected by specific targets like SDG 6.1–4 (clean water and sanitation) and 15.1–3 (life on land), as well as 7.2–3 (affordable and clean energy) that are strongly linked with 13.1 (climate action). We showed also that the strongest interconections between SDG and WEFE are present for the categories of renewable energy system (RED and REW). However, the analysis showed that there is a tendency to focus on a specific sector (e.g. agriculture) and that the good nexus practices implementation is not enough to understand the achievement and progress towards the SDGs. For that reason, we recommended that a more holistic nexus approach including end of supply chain options should be systematically integrated into the project design or evaluation.

## Highlights

• 29 case studies of WEFE nexus implementation were analysed in the Mediterranean area.
• An analytical framework was developed investigating the impacts of 15 SDGs.
• Economic, societal, and environmental perspectives were investigated.
• The magnitude of interconnection of nexus pillars with SDGs was explicitly quantified.
• Six types of technologies were assessed in achieving the SDG targets and WEFE nexus.
• The analysis showed the need of a more holistic nexus approach integrated into the project design.

## 1 Introduction

The WEFE nexus describes the close interlinkages of the water, energy, and food sectors, and details how they depend and potentially impact ecosystems (e.g. freshwater, forests, wetlands and grasslands). These mutual interlinkages define the Water-Energy-Food-Ecosystems (WEFE) nexus, four elements which are crucial to achieving human well-being, poverty reduction and sustainable socio-economic development (Barchiesi et al., 2018; Bervoets et al., 2018).

The nexus concept has been widely promoted in policies since 2011, and albeit there is an open debate regarding its precise meaning and application, the “nexus thinking” is a common fundamental agreement (Simpson and Jewitt, 2019a). The term “nexus thinking” means looking at water, food, energy, and ecosystem behaviour simultaneously, rather than look at any of them in individually. This is a holistic way of thinking that deems long-term implications across the four nexus pillars balancing at the same time social, economic and environmental objectives (Taylor-Wood and Fuller, 2017). However, the irreplaceable foundation of the nexus is to achieve adequate resource security for all while protecting the natural environment. Liu et al. (2018) pointed out that no studies have clearly showed how the WEFE nexus approach can contribute directly or indirectly to the progress and achievement of all SDGs. Accordingly to Rasul (2016), the success in achieving SDGs is strongly related to ensure the sustainable use and management of water, energy, food, and ecosystems. In other words, a nexus approach can improve water, energy, food security and the functionality of ecosystems by increasing the efficiency of use of resources, reducing trade-offs, strengthening synergies, as well as enhancing governance across different sectors (e.g. agricultural and industrial). In Europe this was recently consolidated by the European Commission through its launch of the European Green Deal (EC, 2019), a new growth strategy to ensure sustainability and competitiveness from an economic point of view.

The Mediterranean region is one of the most vulnerable regions in the world where we observe a large spectrum of problematic issues ranging from water pollution (Malagó et al., 2019) and natural resource degradation to water scarcity, large amounts of food loss and waste and increasing demand for energy and food (Markantonis et al., 2019). Here, the application of the WEFE nexus approach will facilitate to alleviate these issues and help achieve SDGs.

Several studies illustrated the nexus concept (Albrecht et al., 2018; Biggs et al., 2015; Cai et al., 2018; de Grenade et al., 2016; Simpson and Jewitt, 2019b; Wichelns, 2017) and the associated simulation tools (Dai et al., 2018; Endo et al., 2020; Kaddoura and El Khatib, 2017; Wicaksono et al., 2017). Zhang et al. (2018), discussing eight modelling approaches, pointed out that a nexus appropriate method is site-specific and should be selected according to research priorities and aims, scales and data availability. Similarly, Bouraoui and Grizzetti, in Barchiesi et al. (2018), identified four categories of WEFE tools considering their increasing data requirements (i.e. qualitative indicator based methods, hydro-economic modelling, integrated Water-Energy-Food nexus, and operational systems) and showed that the availability of data and their integration remain a strong limiting factor in quantitative assessments of the WEFE nexus.

The state-of-the-art of nexus methods applied in the Mediterranean region reflects this status of heterogeneity. For instance, Laspidou et al. (2019) developed a heuristic algorithm that quantifies the intensity of interlinkages amongst five nexus components (water, energy, food, land use and climate) in Greece, taking into account both direct and indirect interrelationships. They also developed a modelling platform to run nexus scenarios and produce forecasted trends (Laspidou et al., 2020). Daccache et al. (2014) investigated the Water-Energy-Food nexus modelling, mapping and quantifying the links between crop production, irrigation demand and energy consumption in the Mediterranean region. Saladini et al. (2018) developed 12 spatial indicators that describe national and local characteristics of the Food-Water interdependencies in the Mediterranean region (e.g. fertilizer consumption, annual freshwater withdrawals and population with access to safe managed water services). Magagna et al. (2019), focusing on Water-Energy nexus in Europe, showed the need to use and manage energy and water resources simultaneously providing an assessment of technological options to reduce water need for energy in particular in Mediterranean countries that will likely experience increased water scarcity by 2050. Finally, based on stakeholders’ perceptions and experts’ opinions, Martinez et al. (2018) identified the main interconnections within the Water-Energy-Food nexus in Andalusia using fuzzy cognitive maps, while Karabulut et al. (2019) developed several multi-criteria analyses to assess the nexus policy impacts.

What has emerged from the analysis of literature, however, is that there is a lack of studies with concrete nexus implementation practices, with respect to the proposed tools and methods as those explained in Terrapon-Pfaff et al. (2018). As a matter of fact, for the best of our knowledge, few studies report real application of nexus (e.g. Barchiesi et al., 2018; Hoff et al., 2019). This can be explained by the presence of several constraints, such as insufficient incentives, limited vision, knowledge, development and investment, as well as the absence of empirical evidence of the potential benefits of a WEFE nexus approach (Hoff et al., 2019). This was also highlighted by Liu et al. (2020) in the special issue “Food-Energy-Water Nexus for Multi-scale Sustainable Development” underlining the limited effective implementation of nexus approaches due to insufficient understanding of nexus trade-offs amongst science-policy-stakeholder interactions (Liu et al., 2017). In this context, our study contributes by providing a new approach for analysing real case studies of practical nexus implementation following the footsteps of Hoff et al. (2019). The main innovative aspect of this work lies in the development of an analytical framework which has the ability to assess if a good nexus practice implemented in a case study produces a change with respect to the baseline. This change resulting potentially in achieving some of the SDG targets by 2030. Based on expert judgement, we investigated the impacts of changes on SDGs and we quantified the WEFE nexus interconnections linking the four nexus pillars to selected SDGs.

In particular, this study explicitly assessed and quantified how the nexus solutions deployed in 29 case studies in the Mediterranean region contribute to a potential achievement of SDG targets by 2030 with regard to the sustainability from the perspectives of economy, environment and society. Finally, we summarized the main findings, and highlighted the new opportunities and the remaining challenges in implementing the WEFE nexus in the Mediterranean region.

## 2 Materials and methods

### 2.1 The case studies

The European Commission's Joint Research Centre (JRC), the Union for the Mediterranean (UfM) and the Global Water Partnership for the Mediterranean (GWP-Med) joined forces to address the complex WEFE nexus in the Mediterranean region. Following a call for collecting case studies that show the benefits of integrating the “nexus thinking” from economic, societal, and environmental perspectives, around 50 proposals were received and scrutinised (JRC, 2020).

Using a specific questionnaire (Figure S1 in the supplementary material), the owners of case studies were invited to provide: i) the description of the case study before (the baseline) and after the implementation of innovative and integrated solutions in the context of WEFE nexus; ii) the analysis of synergies and trade-offs; iii) the evaluation of the resource use efficiency; iv) specific economic, environmental and social achievements. Through the analysis of these questionnaires, we selected 29 case studies based on three main criteria: i) the completeness of the case study description and data supporting claims; ii) the status of the project (when the status of the project was reported, we selected the completed case study only); iii) existence of interactions between more than two elements of the WEFE nexus.

**Fig. 1:**
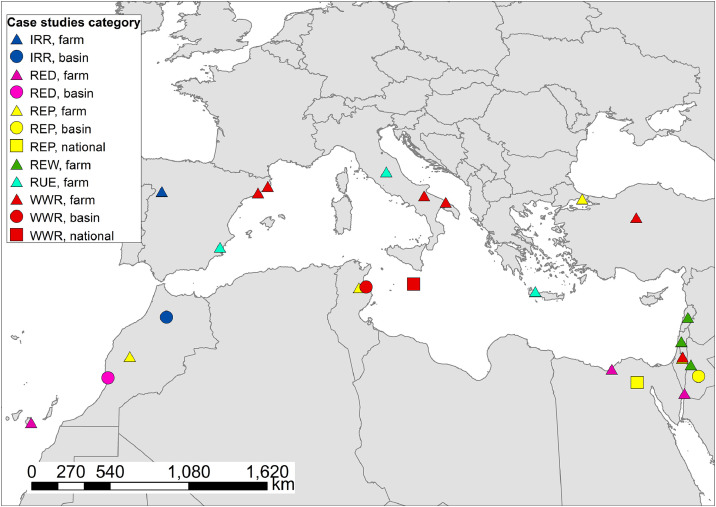
Spatial distribution of collected case studies in the Mediterranean region subdivided by good nexus practices categories and spatial scales (local/farm; basin/region; national). IRR: new techniques of irrigation; RED: use of renewable energy for desalination; REP: use of renewable energy for pumping/distribute water for irrigation or other uses; REW: use of renewable energy for waste water treatment processes; RUE: resources use efficiency; WWR: wastewater reuse.

The selected case studies cover a different range of activities, from farm to national level. Fig. 1 shows the spatial distribution of the selected case studies, and a summary description is reported in Table S1 in the supplementary material.

For the analysis, we classified each case study based on its main good nexus practices implemented as reported in the column “Category” in Table 1. In particular, we identified 6 categories: 1) new techniques of irrigation (IRR); 2) use of renewable energy for desalination (RED); 3) use of renewable energy for pumping/distribution of water for irrigation or other uses (REP); 4) use of renewable energy in wastewater treatment for requirements and/or processes/distribution (REW); 5) resource use efficiency (RUE); 6) wastewater reuse (WWR).

**Table 1:** Summary of case studies collected in the Mediterranean region with different categories of good nexus practices.

Category	Description	Number of case studies
IRR	New irrigation techniques. Modernization of irrigation systems using water-energy saving technologies (e.g. sprinkler system, drip irrigation). The energy supply for the irrigation generally changed from pump units consuming diesel to modern pumping stations supplied by the electricity grid or solar energy	2
RED	Renewable energy for desalination. Solar energy used in desalination plants. In the case studies both solar and on-shore wind power systems are described, as well as the treated water used in agriculture and for fish farming	5
REP	Renewable energy for pumping/distribution of water. Use of renewable energy for pumping system, storage, and distribution. The main uses are for irrigation, for electrification of rural areas, for improving local industrial production (e.g. electric weaving machines as a basis for income generation for women), and for providing electricity to drinking water treatment plants	7
REW	Renewable energy for wastewater. Use of renewable energy for wastewater reuse (e.g. the use of wastewater to irrigate crops, or decorative plants by pumping the treated water using solar power)	4
RUE	Resource use efficiency. Specific applications for resources use efficiency (e.g. development of a biorefinery for citrus waste management, integrated olive trees with cultivation of wild asparagus and free-range chickens, increase soil fertility and reduction of synthetic fertilizers using manure and compost)	3
WWR	Wastewater reuse. Reuse of water from wastewater treatment plants without the support of renewable energy. We also include the case of treated water from desalination without the energy supply from renewable energy	8

The majority of case studies focused on the reuse of water from wastewater treatment plants (8 case studies), followed by the use of renewable energy for pumping water for irrigation and desalinization (7 and 5 cases respectively). Four case studies dealt with the use of renewable energy for wastewater reuse, 3 with the increase of resources use efficiency, and 2 illustrated the implementation of a new irrigation system (Table 1).

### 2.2 The analytical framework

**Fig. 2:**
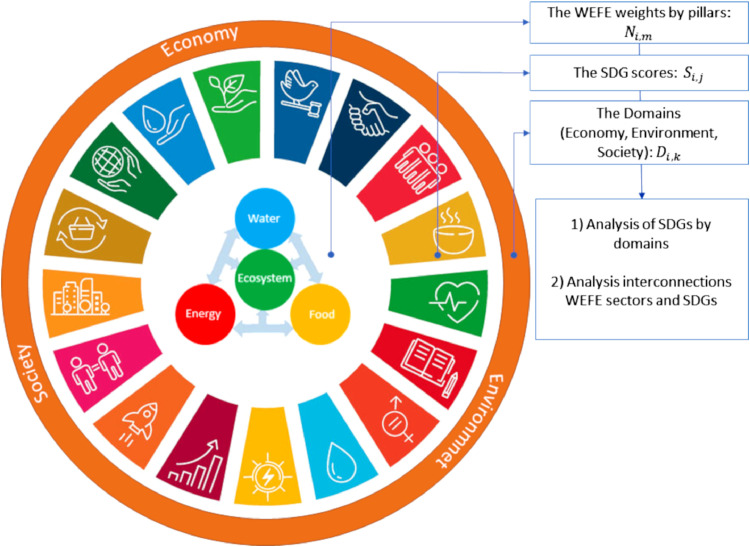
Conceptual framework of nexus approaches. The sub-figure in the middle represents the WEFE nexus; the second sub-figure represents all the SDGs; the external sub-figure shows the three domains that embrace all the SDGs and the WEFE nexus.

The analytical framework we developed is based on three important postulates extracted from Liu et al. (2018): a) “The nexus is directly or indirectly connected with all SDGs”; b) “Nexus approaches promote the achievement of SDGs since the goals are interconnected”; c) “SDG goals are interconnected and linked with the pillars of a particular nexus”. The framework is shown conceptually in Fig. 2.

Based on these postulates, each case study was analysed including the identification of the main technical solution adopted (good nexus practices). Then we selected the main representative SDG targets that can potentially be affected or not. After that we assigned to each of them their economic, environmental and/or social domain. At the same time, since SDG goals are interconnected and linked with the pillars of a particular nexus (Liu et al., 2018), each pillar of the nexus was assigned a numerical value corresponding to the magnitude of interconnection with SDG. Each case study was assigned a positive (+1), negative (−1) or zero score considering how the nexus solutions contribute to a potential achievement of SDG. In particular, the scores were assigned based on a common question for each SDG: “Does the good nexus practice implemented in this case study provide a change for a potential achievement of this SDG target?”

The scores were assigned by the study authors who have different backgrounds from engineering and natural sciences to economic and social academic disciplines. It is noteworthy that, as pointed out by Knol et al. (2010), there is no absolute guideline on which to base the number of experts. According to Knol et al. (2010) at least six experts should be included, while including more than 12 experts will not bring any additional significant benefit.

The developed matrices were used to investigate the impact of SDG potential achievements on economy, environment and society by good nexus practices categories. Finally, we evaluated the WEFE nexus status, highlighting if a balance between the different pillars is achieved.

#### 2.2.1 The SDG matrix

To understand the impact of each case study on the SDGs we developed a matrix approach similar to that provided by Hoff et al. (2019). First, since each SDG has several targets that need to be achieved for each goal, we identified 15 sustainable targets (Table 2) that can potentially be achieved by each case study. Some of them correspond to a single SDG target, others are a combination of multiple SDG targets that have the same potential main objective that could be achieved or not.

**Table 2:** Description of selected SDGs and the corresponding domains retrieved from https://www.un.org/sustainabledevelopment/sustainable-development-goals/ where detailed description of each target is reported.

SDG targets	Domains	Description
Decrease inequality and reduce poverty (SDG 1/SDG 4/SDG 5/SDG 10)	Economy /Society	Guarantee inclusive, equitable quality education and learning opportunities for all (decrease all gender and other inequalities)
Food security and livelihoods (SDG 2.1/2.2/2.3)	Society	End hunger, end all forms of malnutrition and ensure food for all people, with particular attention to the poorest and infants
Human well-being (SDG 3.9)	Economy /Society	Promote well-being and guarantee healthy lives
Water supply (SDG 6.1)	Economy/Environment/Society	Achieve universal and equitable access to safe and affordable drinking water for all
Sanitation (SDG 6.2)	Economy /Society	Achieve access to adequate and equitable sanitation and hygiene for all and end open defecation, paying special attention to the needs of women and girls and those in vulnerable situations
Water quality (SDG 6.3)	Economy/Environment/Society	Improve water quality by reducing pollution, eliminating dumping and minimizing release of hazardous chemicals and materials, halving the proportion of untreated wastewater and substantially increasing recycling and safe reuse globally
Water efficiency (SDG 6.4)	Economy/Environment/Society	Increase water use efficiency and ensure freshwater supply. In particular, this target addresses the issue of water scarcity and the importance of increasing water-use efficiency
Energy supply (SDG 7.1)	Economy/Environment/Society	Ensure universal energy access (i.e. access to electricity) to affordable, reliable and modern energy services
Energy efficiency and increased share in renewables (SDG 7.2, SDG 7.3, /7a and b)	Economy/Environment	Increase substantially the share of renewable energy in the global energy mix thus taking urgent action to combat climate change and its impact. Ensure access to affordable, reliable, sustainable, modern and clean energy services improving the efficiency. In particular, facilitate access to renewable energy and cleaner fossil fuel technology
Employment opportunities (SDG 8.2/8.3/8.5/8.9)	Economy/Environment/Society	Promote sustainable economic growth in accordance with national circumstances, achieving higher levels of economic productivity through e.g. technological upgrading. Promote employment and decent work for all and promote sustainable tourism
Resource use efficiency (SDG 8.4/12.2)	Economy/Environment/Society	More efficient use of resources in terms of consumption and production (e.g. reduce ecological footprint)
Reducing waste (SDG 12.5)	Economy/Environment	Substantially reduce waste generation through prevention, reduction, recycling and reuse
Climate resilience (SDG 13.1)	Economy/Environment/Society	Strengthen resilience and adaptive capacity to climate-related hazards and natural disasters
Conserve and sustainably use the oceans, seas and marine resources (SDG 14.1)	Environment	Prevent and significantly reduce marine pollution of all kinds, in particular from land-based activities, including marine debris and nutrient pollution
Protect and restore terrestrial ecosystems (SDG 15.1/15.3/15.5)	Environment	Ensure the conservation, restoration and sustainable use of terrestrial and inland ecosystems, combat desertification, and take urgent action to reduce the degradation of natural habits

Each case study was evaluated, assigning to each target a score of one of the following values: −1, 0, and +1. A value of −1 indicates a potential non-achievement of the SDG target. The value +1 is assigned for a potential achievement of the SDG target. A zero score was assigned if the case study is not connected to SDG targets.

The scores were first assigned independently by each author, and then compared. The scores with agreement between authors were immediately accepted, while for those without direct agreement, further investigation was undertaken, requesting clarification from the case study owner, as well as further exploration of the literature.

The final matrix is the S_i,j_ “the case studies matrix”:

$$S_{i,j}=\left[\begin{array}{ccccc} S_{1,1} & S_{1,2} & S_{1,3} & \ldots & S_{1,29}\\ S_{2,1} & S_{2,2} & S_{2,3} & \ldots & S_{2,29}\\ S_{3,1} & S_{3,2} & S_{3,3} & \ldots & S_{3,29}\\ \ldots & \ldots & \ldots & \ldots & \ldots \\ \ldots & \ldots & \ldots & \ldots & \ldots \\ \ldots & \ldots & \ldots & \ldots & \ldots \\ \ldots & \ldots & \ldots & \ldots & \ldots \\ S_{15,1} & S_{15,2} & S_{15,3} & ... & S_{15,29} \end{array}\right]$$

Where i represents the SDG targets, j the case studies, and S_i,j_ is the score that ranges from -1 to +1. This matrix is further explained in the supplementary material.

#### 2.2.2 The assignation of domains

Each target has an impact on economy, environment and society (hereafter “the domains”) and they are interconnected (Giddings et al., 2002). For that reason, based on expert judgement and the analysis presented by Dohlman and Advisor (2014), we assigned to each target the relevant domain (*k* = 1 for economy, *k* = 2 for environment, and *k* = 3 for society). Mathematically this is interpreted as a matrix D_i,k_ “the domain matrix” where 1 identifies the evidence of the impact of each SDG (i) on the domain (k), and zero indicates the lack of influence:

$$D_{i,k}=\left[\begin{array}{ccc} 1 & 0 & 1\\ 0 & 0 & 1\\ 1 & 0 & 1\\ 1 & 1 & 1\\ 1 & 0 & 1\\ 1 & 1 & 1\\ 1 & 1 & 1\\ 1 & 1 & 1\\ 1 & 1 & 0\\ 1 & 1 & 1\\ 1 & 1 & 1\\ 1 & 1 & 0\\ 1 & 1 & 1\\ 0 & 1 & 0\\ 0 & 1 & 0 \end{array}\right]$$

Where i represents the SDG targets by rows, and k is the domain by column.

#### 2.2.3 The WEFE weighting

Since SDG targets are interconnected and linked with the pillars of a particular nexus (Liu et al., 2018), we quantified the WEFE nexus interconnections linking each pillar to each SDG target assigning a weight to each combination of SDG target and WEFE pillar. The weight ranges from 1 to 3, where 1 and 3 mean low and high interconnection, respectively. The resulting matrix is N_i,m_ “the nexus matrix”:

$$N_{i,m}=\left[\begin{array}{cccc} 3 & 3 & 3 & 1\\ 2 & 2 & 3 & 1\\ 2 & 1 & 2 & 2\\ 3 & 2 & 1 & 1\\ 3 & 2 & 1 & 2\\ 3 & 1 & 1 & 3\\ 3 & 3 & 2 & 2\\ 2 & 3 & 2 & 1\\ 2 & 3 & 2 & 3\\ 2 & 3 & 2 & 2\\ 2 & 2 & 3 & 3\\ 3 & 3 & 3 & 3\\ 2 & 2 & 2 & 3\\ 2 & 1 & 1 & 3\\ 2 & 1 & 2 & 3 \end{array}\right]$$

Where i represents the SDG target by rows, and m the WEFE pillar by column (*m* = 1 Water, *m* = 2 Energy, *m* = 3 Food, *m* = 4 Ecosystem).

#### 2.2.4 The analysis of SDGs in the perspective of domains and nexus

Knowing S_i,j_, D_i,k_ and N_i,m_, we calculated the impacts of SDG targets on each domain and finally we explicitly quantified the SDG interconnections with WEFE pillars.

The impact of the potential achievement (or not) of SDG targets in each domain was quantified for each case study with the Hadamard Product (element-wise multiplication) between the vector of the selected domain and the vector of case study. For instance, the impact on economy (*k* = 1) of case study *j* = 1 was calculated as follows:

$$DS_{i,j=1,k=1}=\left[\begin{array}{c} D_{1,1}\\ D_{2,1}\\ D_{3,1}\\ \ldots \\ \ldots \\ \ldots \\ \ldots \\ \ldots \\ \ldots \\ \ldots \\ \ldots \\ \ldots \\ \ldots \\ \ldots \\ D_{15,1} \end{array}\right]⊙\left[\begin{array}{c} S_{1,1}\\ S_{2,1}\\ S_{3,1}\\ \ldots \\ \ldots \\ \ldots \\ \ldots \\ \ldots \\ \ldots \\ \ldots \\ \ldots \\ \ldots \\ \ldots \\ \ldots \\ S_{15,1} \end{array}\right]=\left[\begin{array}{c} D_{1,1}\cdot S_{1,1}\\ D_{2,1}\cdot S_{2,1}\\ D_{3,1}\cdot S_{3,1}\\ \ldots \\ \ldots \\ \ldots \\ \ldots \\ \ldots \\ \ldots \\ \ldots \\ \ldots \\ \ldots \\ \ldots \\ \ldots \\ D_{15,1}\cdot S_{15,1} \end{array}\right]$$

The comprehensive impact of good nexus practices of a selected case study (e.g. *j* = 1) on potential SDG achievement by domains (e.g. economy, *k* = 1) was calculated as follows:

$$\sum_{i}^{15}DS_{i,j=1,k=1}$$

The same procedure was applied for the other domains.

The evaluation of interconnections between WEFE nexus pillars and SDGs were calculated for each case study multiplying (element-wise) the vector of the selected pillar from N_i,m_ and the vector of case study from S_i,j_. For instance, the links between Water pillar (*m* = 1) and SDG targets of case study *j* = 1 was calculated as follows:

$$NS_{i,j=1,m=1}=\left[\begin{array}{c} N_{1,1}\\ N_{2,1}\\ N_{3,1}\\ \ldots \\ \ldots \\ \ldots \\ \ldots \\ \ldots \\ \ldots \\ \ldots \\ \ldots \\ \ldots \\ \ldots \\ \ldots \\ N_{15,1} \end{array}\right]⊙\left[\begin{array}{c} S_{1,1}\\ S_{2,1}\\ S_{3,1}\\ \ldots \\ \ldots \\ \ldots \\ \ldots \\ \ldots \\ \ldots \\ \ldots \\ \ldots \\ \ldots \\ \ldots \\ \ldots \\ S_{15,1} \end{array}\right]=\left[\begin{array}{c} N_{1,1}\cdot S_{1,1}\\ N_{2,1}\cdot S_{2,1}\\ N_{3,1}\cdot S_{3,1}\\ \ldots \\ \ldots \\ \ldots \\ \ldots \\ \ldots \\ \ldots \\ \ldots \\ \ldots \\ \ldots \\ \ldots \\ \ldots \\ N_{15,1}\cdot S_{15,1} \end{array}\right]$$

Thus, for instance, the comprehensive evaluation of interconnections of a WEFE pillar (e.g. Water, *m* = 1) and SDG targets for a selected case study was calculated as follows:

$$\sum_{i}^{15}NS_{i,j=1,m=1}$$

The same procedure was applied to the other pillars.

The results by case study, both by domains and WEFE nexus, were aggregated by good nexus practices categories considering the number of case studies and the maximum score expected for each category.

#### 2.2.5 The visualization of the results

The comprehensive impact of good nexus practices on a potential SDG achievement by domains and interconnections between WEFE nexus pillars and SDGs are visualized using a metre score and a polar plot, respectively.

In particular, the hypothesis under our metric implies that if all selected SDG targets resulted potentially achieved due to a change from the baseline for nexus practices implementation, each pillar of the nexus should be numerically close to the others. Visually this can be illustrated in a simple polar plot. The area covered by each quarter of the circle is a measure of the overall interconnection of SDG targets and WEFE pillars. Each quarter represents a pillar of the nexus. The results obtained in each quarter were scored out of 100, where 0 indicates the absence of interconnection between SDG targets and WEFE pillars, while 100 indicates a maximum interconnection with the particular nexus pillar.

This is not a conventional WEFE plot, e.g. radial plot often used in the nexus approach, that quantifies for example “how much Water-Energy-Food-Ecosystem nexus is impacted?”. Here instead, we address the question: “What is the magnitude of interconnection between SDG targets and WEFE pillars?”. The maximum score (100) represents a fully sustainable system.

This procedure was automated and the results visualized using R software (R Core Team, 2011), developing a script that can be used for other case studies.

## 3 Results and discussion

### 3.1 The potential SDG achievements in the perspectives of economy, environment and society

The potential achievement of SDG targets by the reviewed case studies has generally demonstrated positive impacts on economy, environment, and society (Supplementary material, Table S3).

**Fig. 3:**
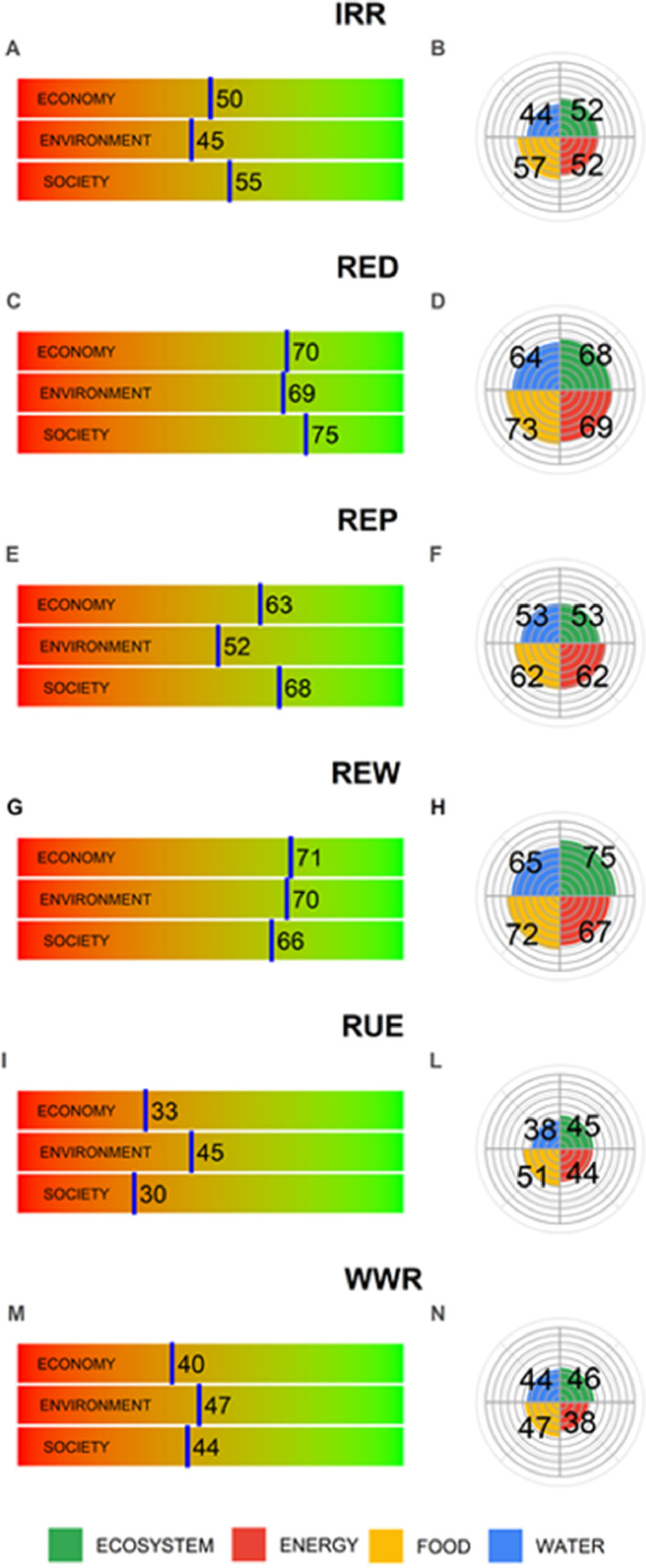
Impacts of SDG achievements on economy, environment and society (left side), and on the WEFE nexus (right side) by category of good nexus practices.

Analysing the results achieved under good nexus practices categories (Fig. 3, left column), it is noticeable that RED (renewable energy for desalinization) and REW (renewable energy in wastewater treatment) have the highest scores in all domains compared to the other categories. In particular, the final score is around 70 for both environment and economy.

This can be explained by the fact that SDG 7 (affordable and clean energy) is fundamentally linked to all other SDGs (Buonocore et al., 2019). As matter of fact, the use of clean energy helps in addressing climate change and ensure healthy lives (SDGs 3 and 13), as well as in preserving life in water and on land (SDGs 6, 14, and 15) providing clean water and sanitation (Fuso Nerini et al., 2018; McCollum et al., 2018).

The impact on society is significant for RED since the water scarcity situation in some countries in the southern Mediterranean is particularly affected by the limitations on drinking water availability. Desalinization of seawater using renewable energy can improve people's socio-economic status, in particular for women, by reducing the time and effort involved in domestic responsibilities and alleviating the health risks associated with currently limited water availability and quality. The improved technology can be a win-win solution at larger scales. For example, it was recently demonstrated that PV-based RO (photo-voltaic powered reverse osmosis desalination) can provide desalinated water for up to 200 million people in the Mediterranean region (Ganora et al., 2019) with a PV installed capacity of 14.2–28.4 GW.

The REP (renewable energy for pumping water for irrigation or other uses) has lower scores than RED and REW, in particular for environment (Fig. 3e). This can be explained by the fact that replacing fossil fuel with solar energy for pumping irrigation, the operational costs for energy are reduced for the user, which may in turn result in overexploitation of water resources (e.g. groundwater). This effect, known as the “rebound effect” in Hoff et al. (2019), was also investigated in depth by Gasparatos et al. (2017), who showed the main impacts on ecosystem and biodiversity of different renewable energy systems, including solar, wind, hydro, ocean, geothermal and bioenergy. For instance, the authors pointed out that solar energy infrastructure can modify a significant amount of territory, fragment habitats and lead to a direct mortality of birds for collisions and burning from solar rays. In addition, solar energy systems are treated with dust suppressants and herbicides, and there is the risk of pollution of water bodies via wash off. In addition, the local microclimates can be affected by solar structures. Concerning wind energy installation, the authors reported that it can cause a small loss of habitats, disrupt the migratory routes of some bird species and there is the risk of collisions with wind turbines.

The “rebound effect” also impacts the IRR category (new irrigation systems), in particular the environment domain with a score of 45/100 (Fig. 3a). Albeit the new irrigation systems allow improving the water and energy use efficiency in agriculture (maximizing food production with less use of water and energy) there is the risk that farmers use the savings to expand irrigation areas and to adopt more intensive crop production (Hoff et al., 2019) resulting in a bigger threat to the environment through the loss of ecosystems services and increased geenhouse gas emissions (Burkart, 2007). Economy and society are less positively impacted, scoring 50/100 and 55/100, respectively, with respect to renewable energy categories. Two major factors can explain the lower positive impacts. First, although improved water efficiency means farmers save on groundwater pumping costs, there is the risk of non-equitable sharing of water between farmers due to unequal access to water (Bell et al., 2016). Second, the improvement of the water-efficient irrigation system also requires a change of cropping system encouraging farmes to plant more water resilient crops, as well as optimum irrigation rates and scheduling over a growing season (Levidow et al., 2014). However, any efforts to improve the irrigation systems needs the support of policies and suitable assistance to farmers in order to develop sustainable environmental behaviours and maintain their economical status (Pereira et al., 2012).

Case studies in the WWR category (wastewater reuse, Fig. 3m), if not supported by renewable energy sources, have low economic (40/100), social (44/100) and environmental beneficial impacts (47/100). The main reason is that wastewater treatment plants use a significant amount of energy with the accompanying greenhouse gas emissions. While the reuse of treated effluents can help reduce the gap between water demand and supply, many challenges remain including the public acceptance of wastewater reuse (Caucci and Meyer, 2017; Fielding et al., 2019).

Similar findings were obtained for the RUE (resource use efficiency) category (Fig. 3i), for which economy and society achieved the lowest scores of 33/100 and 30/100, respectively. This can be explained by the fact the case studies under this category are local examples of reuse of resources (e.g. integrated olive trees with cultivation of wild asparagus and free-range chickens) with a limited scope of people who could obtain benefit from the proposed nexus solution, and thus few SDG targets achieved.

### 3.2 The SDGs interconnections with WEFE pillars

The achievement of SDG targets also has significant impacts on each pillar of the WEFE nexus due to existing interconnections (Liu et al., 2018). The hypothesis under our analytical framework implies that if all selected SDG targets are achieved, each pillar of the nexus should be in equilibrium with the others, thus obtaining close scores. In addition, the higher the score, the stronger the SDG-WEFE pillars interconnections.

Analysing the results obtained by good nexus practices categories (Fig. 3, right column), it is noteworthy that strong interconnections are present in the RED and REW systems, highlighting again the insightful role of SDG 7 and its influence on all other SDGs. The renewable energy categories RED and REW resulted in the most sustainable solutions: all the pillars have the highest scores, around 70/100 for food, energy, and ecosystems, and 65/100 for water. The significant positive impacts of SDG achievements on Ecosystems and Food were noticeably well balanced and maximized.

However, the Water pillar in all categories scores the lowest value, albeit the magnitude is similar to the others. This means that all the specific categories proposed in our case studies need more consideration of water resource management whereby all sectors should be addressed contextually with all their needs. In other words, many of the case studies focus their attention on a specific sector, for example, agriculture, without investigating the effects of the use of the water resources for other activities that coexist in the same area. This is an important issue in a water scarce zone like the Mediterranean region where technical solutions alone cannot provide the increasing population with drinking water supply, food needs and proper environmental management (Hamdy, 2001).

However, the Energy pillar is also slightly penalized for the same reason as Water, since many case studies focus their attention on energy supply to a specific sector, for example, energy supply for water needs in agriculture and not for increasing the proportion of population with access to electricity (SDG 7.1).

This also confirms the general belief that SDGs 6 and 7 have the highest number of potential synergies (Buonocore et al., 2019; Fader et al., 2018). Therefore, achieving SDG water and energy targets will allow achieving other targets, and thus a more sustainable WEFE nexus.

The nexus for REP (use of renewable energy for pumping and distribution water) is less sustainable, mainly due to the difficulty in achieving many SDG targets related to Environment (Table 2). Similar results were obtained for IRR, while the poorest situation was observed for the RUE and WWR category where Energy and Water pillars are unbalanced compared to Food and Ecosystems.

### 3.3 Towards a more holistic nexus approach

What has emerged from the analysis of the case studies is that they focus mainly on the use of suitable technologies and/or practices, but the nexus approach involves more than technical and economic efficiency. The complex links between the four pillars of WEFE nexus needs to be systematically integrated into the project design or evaluated using a more holistic approach (Terrapon-Pfaff et al., 2018). A holistic approach which considers all stakeholders, policy makers, footprints of water production, distribution, and allocation between sectors, such as energy costs, is required for long-term and sustainable management decisions (Zarei, 2020). Vanham et al. (2019) pointed out how the environmental footprints are insightful indicators that provide essential information for an analysis of the WEFE nexus because they quantify pressures along the whole supply chain, up to the consumer level. In this context, the need of identifying specific end of supply chain solutions (e.g. diet behaviour, reduction of food losses and waste along the supply chain) is fundamental for reducing the effects of consumptive and degradative resources utilization. For instance, Vanham (2013) and Vanham et al. (2013b, 2013a) showed that improving diets with a healthier and vegetarian foods in Europe lead to a saving of water between 23%–38% since the consumption of animal products accounts for the largest share of water footprint (45%).

However, a spatial-temporal monitoring at all stages along the supply chain is necessary. As a matter of facts, Vanham et al. (2018) highlighted that to monitor water scarcity it is necessary to take into account the strong spatial and temporal variability in water availability, as well as the use of environmental flow. They recommended to use both annual and monthly values, as well as a high spatial resolution of water resources. In this context, the modelling frameworks and remote sensing data are insightful instruments for identifying hot spot areas where planning specific interventions toward the achievement of the SDGs and doing scenarios analysis. In this context Giupponi and Gain (2017) developed a tool to monitor progress and help better resource management to achieve the sustainable goals. Recently, Xu et al. (2020) developed a systematic method for assessing spatial-temporal progress towards achieving the SDGs in China. The authors showed the gaps in sustainable development between western and eastern China and how the new technologies improved social aspects (e.g. education and healthcare) but also created new environmental problems (e.g. water pollution and land degradation). Schmidt-Traub et al. (2017) highlighted also how good data and clear metrics are critical for each country to define a clear baseline of the SDG achievements and track their progress.

## 4 Conclusions

The analytical framework proposed in this study is able to identify and balance both strengths and weaknesses of good nexus practices implemented in each case study and each category, and to give an overall picture of the current status of the WEFE nexus in selected Mediterranean regions. However, the method is not able to track the progress on SDGs achievement. In addition the differences between the spatial scales of application (e.g. farm level and basin scale) are not captured due to the limited spatial information provided. This can increase the risks of unsustainable use of resources because actions taken at such different scales are not well aligned. As a consequence, in addtion to the newly developed framework, we recommend to integrate systematically into the project design of a case study a holistic approach including end of supply chain options supported by spatial-temporal high resolution datasets, as well as an integrated modelling to track progress and do scenarios.

## 5 CRediT author statement

**Anna Malagó**: Conceptualization, Methodology, Software, Validation, Formal Analysis, Investigation, Data Curation, Writing - Original Draft, Writing - Review & Editing, Visualization **Sara Comero**: Conceptualization, Methodology, Software, Validation, Formal Analysis, Investigation, Data Curation, Writing - Review & Editing, Visualization **Fayçal Bouraoui**: Conceptualization, Methodology, Validation, Investigation, Resources, Writing - Review & Editing, Supervision **Cevza Melek Kazezyılmaz-Alhan**: Validation, Investigation, Resources, Writing - Review & Editing **Bernd Manfred Gawlik**: Conceptualization, Methodology, Validation, Investigation, Resources, Writing - Review & Editing, Supervision, Project administration **Peter Easton:** Investigation, Resources, Writing - Review & Editing **Chrysi Laspidou**: Conceptualization, Resources.

## Declaration of Competing Interest

The authors declare that they have no known competing financial interests or personal relationships that could have appeared to influence the work reported in this paper.

## References

[bib0001] Albrecht T.R., Crootof A., Scott C.A. (2018). The Water-Energy-Food Nexus: a systematic review of methods for nexus assessment. Environ. Res. Lett..

[bib0002] Barchiesi S., Carmona-Moreno C., Dondeynaz C., Biedler M. (2018). Proceedings of the Workshop on Water-Energy-Food-Ecosystems (WEFE) Nexus and Sustainable Development Goals (SDGs).

[bib0003] Bell A.R., Ward P.S., Shah M.A.A. (2016). Increased water charges improve efficiency and equity in an irrigation system. Ecol. Soc..

[bib0004] Bervoets J., Eveillé F., Thulstru A. (2018). Strengthening the Water-Food-Energy-Ecosystems (WFEE) Nexus.

[bib0005] Biggs E.M., Bruce E., Boruff B., Duncan J.M.A., Horsley J., Pauli N., McNeill K., Neef A., Van Ogtrop F., Curnow J., Haworth B., Duce S., Imanari Y. (2015). Sustainable development and the water-energy-food nexus: a perspective on livelihoods. Environ. Sci. Policy.

[bib0006] Buonocore J.J., Choma E., Villavicencio A.H., Spengler J.D., Koehler D.A., Evans J.S., Lelieveld J., Klop P., Sanchez-Pina R. (2019). Metrics for the sustainable development goals: renewable energy and transportation. Palgrave Commun.

[bib0007] Burkart M.R. (2007). Diffuse pollution from intensive agriculture: sustainability, challenges, and opportunities. Water Science and Technology.

[bib0008] Cai X., Wallington K., Shafiee-Jood M., Marston L. (2018). Understanding and managing the food-energy-water nexus – opportunities for water resources research. Adv. Water Resour..

[bib0009] Caucci S., Meyer K. (2017). Your Future Food Will Be Grown With Waste Water [WWW Document]. https://theconversation.com/your-future-food-will-be-grown-with-waste-water-74009.

[bib0010] Daccache A., Ciurana J.S., Rodriguez Diaz J.A., Knox J.W. (2014). Water and energy footprint of irrigated agriculture in the Mediterranean region. Environ. Res. Lett..

[bib0011] Dai J., Wu S., Han G., Weinberg J., Xie X., Wu X., Song X., Jia B., Xue W., Yang Q. (2018). Water-energy nexus: a review of methods and tools for macro-assessment. Appl. Energy.

[bib0012] de Grenade R., House-Peters L., Scott C.A., Thapa B., Mills-Novoa M., Gerlak A., Verbist K. (2016). The nexus: reconsidering environmental security and adaptive capacity. Curr. Opin. Environ. Sustain.

[bib0013] Dohlman E., Advisor S. (2014). Policy Coherence for Sustainable Development in the Post-2015 Framework. Slide 4 by Amb. Csaba Kőrösi, PR of Hungary to UN: “From SDGs to Post-2015 Agenda” at the OECD in Paris on October 7th, 2014.

[bib0014] EC (2019). The European Green Deal. https://ec.europa.eu/info/sites/info/files/european-green-deal-communication_en.pdf.

[bib0015] Endo A., Yamada M., Miyashita Y., Sugimoto R., Ishii A., Nishijima J., Fujii M., Kato T., Hamamoto H., Kimura M., Kumazawa T., Qi J. (2020). Dynamics of water–energy–food nexus methodology, methods, and tools. Curr. Opin. Environ. Sci. Heal..

[bib0016] Fader M., Cranmer C., Lawford R., Engel-Cox J. (2018). Toward an Understanding of Synergies and Trade-Offs Between Water, Energy, and Food SDG Targets. Front. Environ. Sci..

[bib0017] Fielding K.S., Dolnicar S., Schultz T. (2019). Public acceptance of recycled water. Int. J. Water Resour. Dev..

[bib0018] Fuso Nerini F., Tomei J., To L.S., Bisaga I., Parikh P., Black M., Borrion A., Spataru C., Castán Broto V., Anandarajah G., Milligan B., Mulugetta Y. (2018). Mapping synergies and trade-offs between energy and the Sustainable Development Goals. Nat. Energy.

[bib0019] Ganora D., Dorati C., Huld T.A., Udias A., Pistocchi A. (2019). An assessment of energy storage options for large-scale PV-RO desalination in the extended Mediterranean region. Sci. Rep..

[bib0020] Gasparatos A., Doll C.N.H., Esteban M., Ahmed A., Olang T.A. (2017). Renewable energy and biodiversity: implications for transitioning to a Green Economy. Renew. Sust. Energy Rev..

[bib0021] Giddings B., Hopwood B., O'Brien G. (2002). Environment, economy and society: fitting them together into sustainable development. Sustain. Dev..

[bib0022] Giupponi C., Gain A.K. (2017). Integrated spatial assessment of the water, energy and food dimensions of the Sustainable Development Goals. Reg. Environ. Chang..

[bib0023] Hamdy, A., 2001. WATER RESOURCES MANAGEMENT IN THE MEDITERRANEAN COUNTRIES: PRIORITY ACTIONS.MEDIT. This paper is a synthesis of the third chapter of the CIHEAM report 2000 on Agricultural Development and Agrofood policies in the Mediterranean region.

[bib0024] Hoff H., Alrahaife S.A., El Hajj R., Lohr K., Mengoub F.E., Farajalla N., Fritzsche K., Jobbins G., Özerol G., Schultz R., Ulrich A. (2019). A Nexus Approach for the MENA Region—From Concept to Knowledge to Action. Front. Environ. Sci..

[bib0025] JRC (2020). Water-Energy-Food-Ecosystem Nexus in the Mediterranean - Seeking Examples of Best Practices - EU Science Hub [WWW Document]. https://ec.europa.eu/jrc/en/science-update/water-energy-food-ecosystem-nexus-mediterranean-seeking-examples-best-practices.

[bib0026] Kaddoura S., El Khatib S. (2017). Review of water-energy-food Nexus tools to improve the Nexus modelling approach for integrated policy making. Environ. Sci. Policy.

[bib0027] Karabulut A.A., Udias A., Vigiak O. (2019). Assessing the policy scenarios for the Ecosystem Water Food Energy (EWFE) nexus in the Mediterranean region. Ecosyst. Serv..

[bib0028] Knol A.B., Slottje P., Van Der Sluijs J.P., Lebret E. (2010). The use of expert elicitation in environmental health impact assessment: a seven step procedure. Environ. Heal. A Glob. Access Sci. Source.

[bib0029] Laspidou C., Mellios N., Kofinas D. (2019). Towards Ranking the Water–Energy–Food–Land Use–Climate Nexus Interlinkages for Building a Nexus Conceptual Model with a Heuristic Algorithm. Water (Basel).

[bib0030] Laspidou C.S., Mellios N.K., Spyropoulou A.E., Kofinas D.T., Papadopoulou M.P. (2020). Systems thinking on the resource nexus: modeling and visualisation tools to identify critical interlinkages for resilient and sustainable societies and institutions. Sci. Total Environ..

[bib0031] Levidow L., Zaccaria D., Maia R., Vivas E., Todorovic M., Scardigno A. (2014). Improving water-efficient irrigation: prospects and difficulties of innovative practices. Agric. Water Manag..

[bib0032] Liu J., Hull V., Godfray H.C.J., Tilman D., Gleick P., Hoff H., Pahl-Wostl C., Xu Z., Chung M.G., Sun J., Li S. (2018). Nexus approaches to global sustainable development. Nat. Sustain..

[bib0033] Liu J., Scanlon B.R., Zhuang J., O. V. (2020). Food-Energy-Water Nexus for Multi-scale Sustainable Development. Resour. Conserv. Recycl..

[bib0034] Liu J., Yang H., Cudennec C., Gain A.K., Hoff H., Lawford R., Qi J., Strasser L.de, Yillia P.T., Zheng C. (2017). Challenges in operationalizing the water–energy–food nexus. Hydrol. Sci. J..

[bib0035] Magagna D., Hidalgo Gonzále I., Bidoglio G., Peteves S., Adamovic M., Bisselink B., De Felice M., De Roo A., Dorati C., Ganora D., Medarac H., Pistocchi A., Van De Bund W., Vanham D. (2019). Water – Energy Nexus in Europe.

[bib0036] Malagó A., Bouraoui F., Grizzetti B., De Roo A. (2019). Modelling nutrient fluxes into the Mediterranean Sea. J. Hydrol. Reg. Stud..

[bib0037] Markantonis V., Reynaud A., Karabulut A., El Hajj R., Altinbilek D., Awad I.M., Bruggeman A., Constantianos V., Mysiak J., Lamaddalena N., Matoussi M.S., Monteiro H., Pistocchi A., Pretato U., Tahboub N., Tunçok I.K., Ünver O., Van Ek R., Willaarts B., Bülent S., Zakir T., Bidoglio G. (2019). Can the implementation of the Water-Energy-Food nexus support economic growth in the Mediterranean region? The current status and the way forward. Front. Environ. Sci..

[bib0038] Martinez P., Blanco M., Castro-Campos B. (2018). The Water–Energy–Food Nexus: a Fuzzy-Cognitive Mapping Approach to Support Nexus-Compliant Policies in Andalusia (Spain). Water (Basel).

[bib0039] McCollum D., Gomez Echeverri L., Busch S., Pachauri S., Parkinson S., Rogelj J., Krey V., Minx J. (2018). Connecting the sustainable development goals by their energy inter-linkages - IOPscience. Environmental Res. Lett.

[bib0040] Pereira L.S., Cordery I., Iacovides I. (2012). Improved indicators of water use performance and productivity for sustainable water conservation and saving. Agric. Water Manag..

[bib0041] R Core Team, 2011. https://www.r-project.org/ [WWW Document]. URL https://www.r-project.org/ (accessed 4.1.20).

[bib0042] Rasul G. (2016). Managing the food, water, and energy nexus for achieving the Sustainable Development Goals in South Asia. Environ. Dev..

[bib0043] Saladini F., Betti G., Ferragina E., Bouraoui F., Cupertino S., Canitano G., Gigliotti M., Autino A., Pulselli F.M., Riccaboni A., Bidoglio G., Bastianoni S. (2018). Linking the water-energy-food nexus and sustainable development indicators for the Mediterranean region. Ecol. Indic..

[bib0044] Schmidt-Traub G., Kroll C., Teksoz K., Durand-Delacre D., Sachs J.D. (2017). National baselines for the Sustainable Development Goals assessed in the SDG Index and Dashboards. Nat. Geosci..

[bib0045] Simpson G.B., Jewitt G.P. (2019). The water-energy-food nexus in the anthropocene: moving from ‘nexus thinking’ to ‘nexus action. Curr. Opin. Environ. Sustain..

[bib0046] Simpson G.B., Jewitt G.P.W. (2019). The development of the water-energy-food nexus as a framework for achieving resource security: a review. Front. Environ. Sci..

[bib0047] Taylor-Wood, E., Fuller, D., 2017. Nexus thinking for a secure and sustainable future | Opinion | Eco-Business | Asia Pacific.

[bib0048] Terrapon-Pfaff J., Ortiz W., Dienst C., Gröne M.C. (2018). Energising the WEF nexus to enhance sustainable development at local level. J. Environ. Manage..

[bib0049] Vanham D. (2013). The water footprint of Austria for different diets. Water Sci. Technol..

[bib0050] Vanham D., Hoekstra A.Y., Bidoglio G. (2013). Potential water saving through changes in European diets. Environ. Int..

[bib0051] Vanham D., Hoekstra A.Y., Wada Y., Bouraoui F., de Roo A., Mekonnen M.M., van de Bund W.J., Batelaan O., Pavelic P., Bastiaanssen W.G.M., Kummu M., Rockström J., Liu J., Bisselink B., Ronco P., Pistocchi A., Bidoglio G. (2018). Physical water scarcity metrics for monitoring progress towards SDG target 6.4: an evaluation of indicator 6.4.2 “Level of water stress.”. Sci. Total Environ..

[bib0052] Vanham D., Leip A., Galli A., Kastner T., Bruckner M., Uwizeye A., van Dijk K., Ercin E., Dalin C., Brandão M., Bastianoni S., Fang K., Leach A., Chapagain A., Van der Velde M., Sala S., Pant R., Mancini L., Monforti-Ferrario F., Carmona-Garcia G., Marques A., Weiss F., Hoekstra A.Y. (2019). Environmental footprint family to address local to planetary sustainability and deliver on the SDGs. Sci. Total Environ.

[bib0053] Vanham D., Mekonnen M.M., Hoekstra A.Y. (2013). The water footprint of the EU for different diets. Ecol. Indic..

[bib0054] Wicaksono A., Jeong G., Kang D. (2017). Water, energy, and food nexus: review of global implementation and simulation model development. Water Policy..

[bib0055] Wichelns D. (2017). The water-energy-food nexus: is the increasing attention warranted, from either a research or policy perspective?. Environ. Sci. Policy.

[bib0056] Xu Z., Chau S.N., Chen X., Zhang J., Li Yingjie, Dietz T., Wang J., Winkler J.A., Fan F., Huang B., Li S., Wu S., Herzberger A., Tang Y., Hong D., Li Yunkai, Liu J. (2020). Assessing progress towards sustainable development over space and time. Nature.

[bib0057] Zarei M. (2020). The water-energy-food nexus: a holistic approach for resource security in iran, iraq, and turkey. Water-Energy Nexus.

[bib0058] Zhang C., Chen X., Li Y., Ding W., Fu G. (2018). Water-energy-food nexus: concepts, questions and methodologies. J. Clean. Prod..

